# Severe bronchopulmonary dysplasia improved by noninvasive positive pressure ventilation: a case report

**DOI:** 10.1186/1752-1947-5-435

**Published:** 2011-09-06

**Authors:** Christian Mann, Walter Bär

**Affiliations:** 1 Neonatal and Pediatric Intensive Care Unit, Graubuenden Cantonal Hospital, Loestr 170, CH-7000 Chur, Switzerland; 2 College for Intensive Care, Emergency and Anesthesia Nursing, Children's Hospital, University Clinic, Steinwiesstrasse 75, CH-8032 Zuerich, Switzerland

## Abstract

**Introduction:**

This is the first report to describe the feasibility and effectiveness of noninvasive positive pressure ventilation in the secondary treatment of bronchopulmonary dysplasia.

**Case presentation:**

A former male preterm of Caucasian ethnicity delivered at 29 weeks gestation developed severe bronchopulmonary dysplasia. At the age of six months he was in permanent tachypnea and dyspnea and in need of 100% oxygen with a flow of 2.0 L/minute via a nasal cannula. Intermittent nocturnal noninvasive positive pressure ventilation was then administered for seven hours daily. The ventilator was set at a positive end-expiratory pressure of 6 cmH_2_O, with pressure support of 4 cmH_2_O, trigger at 1.4 mL/second, and a maximum inspiratory time of 0.7 seconds. Over the course of seven weeks, the patient's maximum daytime fraction of inspired oxygen via nasal cannula decreased from 1.0 to 0.75, his respiratory rate from 64 breaths/minute to 50 breaths/minute and carbon dioxide from 58 mmHg to 44 mmHg.

**Conclusion:**

Noninvasive positive pressure ventilation may be a novel therapeutic option for established severe bronchopulmonary dysplasia. In the case presented, noninvasive positive pressure ventilation achieved sustained improvement in ventilation and thus prepared our patient for safe home oxygen therapy.

## Introduction

Although there is some evidence that nasal noninvasive ventilation has the potential to reduce the incidence of bronchopulmonary dysplasia (BPD) in preterm newborns [1-5], there have been no studies of nasal noninvasive positive pressure ventilation (NIPPV) in former preterm infants with an established diagnosis of BPD requiring high oxygen concentrations.

The main pathophysiological finding in BPD is a low functional residual capacity accompanied by inefficient gas mixing. Respiratory rate is increased [6]. Small airway function may worsen during the first year [7]. Significant gas trapping is found in some BPD infants [8,9]. We report the response to intermittent nocturnal therapy with nasal NIPPV in an infant with severe BPD.

### Case presentation

Our patient was a male preterm of Caucasian ethnicity, born at 29 weeks and one day gestation by Caesarean section from a spontaneous dichorionic diamniotic twin pregnancy complicated by preterm premature rupture of the membranes with near-total loss of fluid nine days before delivery. His birth weight was 940 g. A chest X-ray showed pulmonary hypoplasia and grade 3 hyaline membrane disease. Surfactant (beractant 100 mg/kg) was given one hour after birth and repeated 24 hours later.

The patient was started on high frequency oscillatory ventilation, with highest mean airway pressure 22 cmH_2_O on day one, and then switched to pressure-controlled synchronized intermittent mandatory ventilation on day 20 (highest peak inspiratory pressure 24 cmH_2_O). Inhaled nitric oxide was delivered for five days in decreasing amounts (starting on day one with 26 ppm).

A left pneumothorax was drained on day four. The clinical course was complicated by ventilator-associated pneumonia on day 15. Tracheal aspirates grew coagulase-negative *Staphylococci *and *Enterobacter cloacae*. Treatment consisted of piperacillin and tazobactam with fusidic acid for two weeks. Extubation was successful on day 26 after a two-day course of dexamethasone. Ventilatory support was continued with nasal continuous positive airway pressure (nCPAP; 8 cmH_2_O). BPD was diagnosed at postmenstrual age 36 weeks. Shortly thereafter, nasal swab cultures from copious upper airway secretions proved colonization with *Stenotrophomonas maltophilia*, *Escherichia coli *as well as *Staphylococcus aureus *which was treated with a two-week course of oral sulfamethoxazole plus trimethoprim and rifampin.

After 10 weeks nCPAP was switched to nasal cannula flow of 2 L/minute with a fraction of inspired oxygen (FiO_2_) of 0.5. Pulse oximetry target was set at arterial oxygen saturation (SaO_2_) ≥ 90%. During subsequent weeks the oxygen concentration had to be increased to a FiO_2 _of 1.0 due to progressive deterioration of gas exchange. At the age of six months our patient was in constant dyspnea and tachypnea. Spontaneous inspiratory time was markedly shortened. Streaky densities and cystic areas on a chest X-ray confirmed the diagnosis of severe BPD. Echocardiography revealed concomitant pulmonary hypertension with a tricuspid regurgitation pressure gradient up to 30 mmHg. The FiO_2 _1.0 requirement created a high risk of urgent reintubation in the event of sudden desaturation. The boy's increasing drive to move around ruled out reintroducing nCPAP.

A ventilator set to NIPPV was installed providing nocturnal ventilatory support for an average of seven hours every night. Ventilator settings are presented in Table 1. For the first 18 days, sedation was provided with chloral hydrate in decreasing amounts from 52 mg/kg to 7 mg/kg per evening dose.

**Table 1:** Ventilator settings for NIPPV

Pressure support	4 cmH_2_O
Positive end-expiratory pressure	6 cmH_2_O
Trigger	1.4 mL/second
Ramp	25 ms
Expiratory trigger sensitivity	10%
Backup respiratory rate	8/minute
Maximum inspiratory time	0.7 seconds

The features of the NIPPV device included a limited dead space, highly sensitive automated circuit leak compensation, and high trigger sensitivity. NIPPV was administered via a nasal mask in a semirecumbent position to enhance air entry into West zones 1 and 2 and to diminish expansion of the radiologically over-distended lung bases.

**Figure 1:**
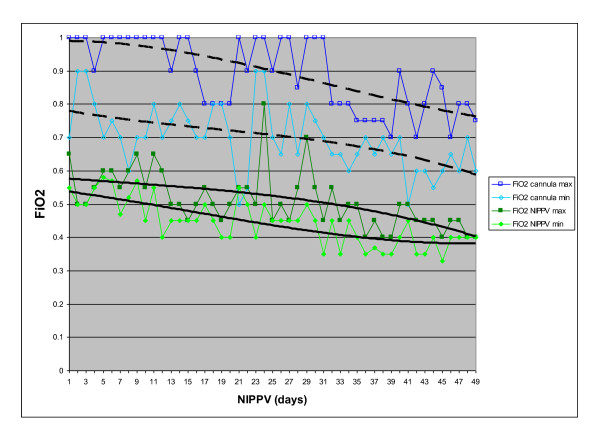
**Decrease in FiO_2 _requirement over seven weeks of nocturnal NIPPV**. Open rectangles and blue lines: oxygen concentrations (maximum and minimum for each day) delivered via nasal cannula during daytime; full rectangles and green lines: oxygen concentrations for nocturnal NIPPV. Oxygen saturation target was set at ≥ 90%.

In the course of seven weeks of intermittent nocturnal NIPPV, the spontaneous respiratory rate decreased from 64 breaths/minute to 50 breaths/minute, morning (post-NIPPV) carbon dioxide dropped from 58 mmHg to 44 mmHg, and--most importantly--nasal cannula maximum FiO_2 _decreased from 1.0 to 0.75 and the minimum FiO_2 _from 0.8 to 0.6 (Figure 1). At this point, NIPPV was stopped and the baby was discharged on home oxygen (flow rate 0.25 L/minute) at the postnatal age of eight months. His weight increased by 200 g per week during NIPPV therapy and reached 7490 g at discharge.

Two intercurrent lower respiratory tract infections were managed on an outpatient basis. Our patient was completely weaned off oxygen nine months after discharge at the age of 17 months.

Neurological examination at the age of one year showed less delay in the mental scale than in the psychomotor scale (Bayley II) with scores of 76 and 56, respectively. Free walking was achieved at 22 months of age.

## Conclusion

The clinical course of this ex-preterm boy suggests that secondary NIPPV therapy has the potential to improve severe BPD. A course of nocturnal intermittent NIPPV in a timely manner (seven weeks) improved ventilation and reduced oxygen need to a degree which provided sufficient safety for subsequent home oxygen therapy. Its positive effect was essential for our patient's discharge after eight months of hospital stay. In terms of practicability, NIPPV was superior to nCPAP in that it reliably avoided hypoventilation when the child initially needed sedation to tolerate a nasal mask.

According to the literature, a bundle of different mechanisms may have contributed to the improvement observed. Synchronized NIPPV is known to increase functional residual capacity [4], enhance ventilation uniformity [5], improve respiratory drive [10,11], lead to greater lung recruitment [12] and decrease inspiratory effort and respiratory work in comparison to continuous flow nCPAP [13,14]. The duration of ventilatory support is shorter with primary use of NIPPV than with nCPAP [15].

We think this observation provides useful information on NIPPV in established BPD before larger randomized studies are performed on this topic. Further studies incorporating lung function tests should identify the level of respiratory support at which the repetitive stimulus of nocturnal NIPPV exerts most of its positive influence. It would be interesting to find out how NIPPV propagates lung remodelling or if it even has the potential to accelerate lung maturation in severe BPD.

## Consent

Written informed consent was obtained from the patient's parents for publication of this case report. A copy of the written consent is available for review by the Editor-in-Chief of this journal.

## Competing interests

The authors declare that they have no competing interests.

## Authors' contributions

WB led the decision to institute NIPPV in this case. WB and CM analyzed and interpreted the patient data. CM reviewed the literature and wrote the manuscript. Both authors read and approved the final manuscript.
